# A rare case report of iatrogenic Cushing syndrome caused by direct anti-hepatitis C virus therapy with sofosbuvir/velpatasvir

**DOI:** 10.1097/MD.0000000000030294

**Published:** 2022-12-23

**Authors:** Bo Ma, Tianling Shang, Jianjie Huang, Zhixin Tu, Yan Wang, Yujin Han, Yang Wang, Xiaoyu Wen, Qinglong Jin

**Affiliations:** a Department of Hepatology, The First Hospital of Jilin University, Changchun, Jilin Province, China; b Department of Neurology, The First Hospital of Jilin University, Changchun, Jilin Province, China.

**Keywords:** adverse drug reaction, case report, iatrogenic Cushing syndrome, sofosbuvir/velpatasvir

## Abstract

Sofosbuvir/velpatasvir (SOF/VEL) is a combination of direct-acting antivirals with pan-genotypic activity that is used to treat chronic hepatitis C virus infection. This was a fixed-dose regimen. SOF is a nucleotide nonstructural 5B polymerase inhibitor and VEL is an nonstructural 5A inhibitor. Side effects of this agent on the endocrine system, particularly iatrogenic Cushing syndrome (ICS), are uncommon. Here, we present a case of ICS with significantly low serum adrenocorticotropic hormone and cortisol levels caused by SOF/VEL.

**Patient concerns:**

A 49-year-old Asian woman with chronic hepatitis C and cirrhosis presented with a round face, fat thickening at the clavicle and back of the neck, mild facial edema, facial congestion, skin ulceration on the hands, central obesity, acne, and general status changes after 3 months of treatment with SOF/VEL (400 mg/dose, 1/day).

**Diagnoses:**

The patient’s serum adrenocorticotropic hormone and cortisol levels dropped significantly, and her normal rhythm vanished, with no visible aberrant lesions on computed tomography or across the abdomen. The patient was diagnosed with ICS.

**Outcomes:**

Symptoms improved after withdrawing SOF/VEL and taking low-dose oral hydrocortisone. Thus, the SOF/VEL was suspected to be an offender. To our knowledge, this is the first time that SOF/VEL has been linked to ICS.

**Lessons:**

Hepatologists and primary care physicians treating hepatitis C virus should be more aware of this uncommon adverse event so that direct-acting antiviral therapy can be stopped sooner if it recurs. The findings of this study emphasize the importance of collaboration between hepatologists and endocrinologists in co-management of complications.

## 1. Introduction

Sofosbuvir/velpatasvir (SOF/VEL) is the first pangenotypic direct-acting antiviral (DAA) licensed for the treatment of hepatitis C virus (HCV) genotypes 1–6 in patients with or without compensated cirrhosis, and it is also the first DAA recommended for the treatment of decompensated cirrhosis when combined with ribavirin.^[1]^ In patients with chronic HCV pangenotypic infection, many studies have demonstrated that SOF/VEL produces a higher sustained virological response in patients with HCV genotypes 1–6.^[2–4]^ There has been no evidence of adverse effects of iatrogenic Cushing syndrome (ICS) in previous clinical studies. Here, we present a rare case of Cushing syndrome caused by SOF/VEL.

## 2. Case report

**Figure 1.**
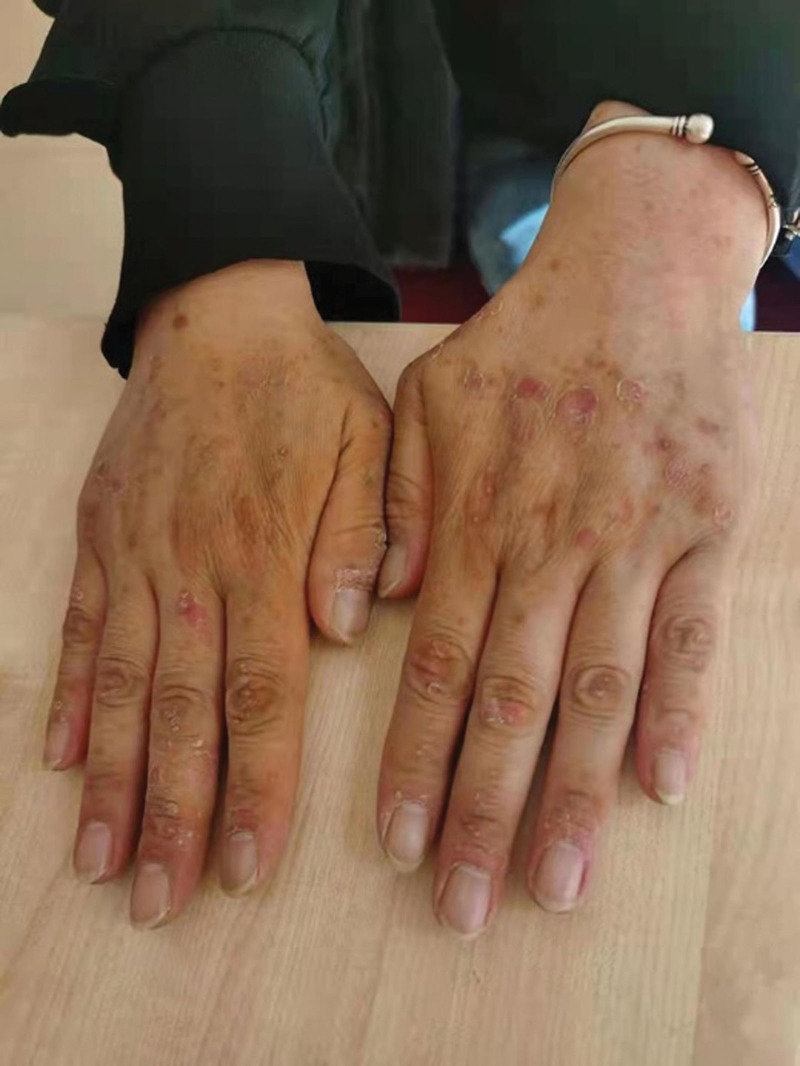
Ulceration of hands after 3 months of SOF/VEL antiviral treatment. SOF/VEL = sofosbuvir/velpatasvir.

A 49-year-old woman was diagnosed with chronic HCV for more than 2 years. The patient visited the Department of Hepatology 2 years prior after being determined to be pathogenically positive for HCV with no obvious etiology. She had a HCV level of 1.10E+006 IU/mL and hepatitis C genotype of 3b. The patient did not receive antiviral treatment until she was readmitted to our department on March 3, 2021. She had psoriasis without a thorough diagnosis and treatment for more than 2 years, but no coronary heart disease, diabetes, hypertension, or other problems. She denied any history of infectious illnesses, such as tuberculosis, any history of trauma or surgery, or any history of medication or food allergies. Prior to treatment with the antiviral drug SOF/VEL, her viral test result was 2.73E+006 IU/mL, and there were no abnormal facial manifestations in February 2021. Three months after receiving SOF/VEL (400 mg/dose, 1/day) and testing negative for the virus, she developed facial edema with bilateral lower limb edema, which gradually worsened but did not cause an alarm. This is followed by shortness of breath after exertion, periodic panic, and palpitations. Examination revealed a round face with thickened supraclavicular and retroclavicular fat, mild facial puffiness, polycythemia, skin breakdown on both hands, centripetal obesity, and acne (Fig. 1).

The results of routine laboratory examinations at admission are presented in Table 1. For the loss of adrenocorticotropic rhythm, the 0am-8am-4pm serum adrenocorticotropic hormone (ACTH) (pmol/L) level was 0.22-0.22-0.22. For the loss of cortisol rhythm, the 0am-8am-4pm serum cortisol (nmol/L) level was 11.2-11.43-19.54. The 24-hour urinary free cortisol was 34.75 nmol/24 hour. Myocardial injury markers, fasting glucose, thyroid hormone, stool examination, coagulation routine, sex hormones, electrocardiography, cardiac ultrasound, cranial computed tomography (CT), and pulmonary CT did not show any significant abnormalities. CT of the entire abdomen revealed liver cirrhosis and a slightly enlarged spleen.

**Table 1:** Routine laboratory workup at admission.

	Laboratory results	Normal range
Hemoglobin (g/L)	129	130–150
White blood cells (10^9^/L)	13.74	3.50–9.50
Red blood cells (10^9^/L)	4.00	4.30–5.80
Lymphocyte percentage (10^9^/L)	10.35	1.10–3.20
Blood platelets (10^9^/L)	104	125–350
Alanine aminotransferase (U/L)	275.2	9–50
Aspartate aminotransferase (U/L)	190.5	15.0–40.0
Gamma-glutamyl transferase (U/L)	318.8	10–60.0
Alkaline phosphatase (U/L)	190.0	45.0–125.0
Cholinesterase (U/L)	2973	4620–11,500
Total bilirubin (mmol/L)	27.4	6.8–30.0
Direct bilirubin (mmol/L)	10.9	0.0–8.6
Albumin (g/L)	28.3	40.0–55.0
Prothrombin activity (%)	122	80–120
Fasting glucose (mmol/L)	7.85	3.9–6.1
Alpha-fetoprotein (ng/mL)	193.50	0–7.0
Carcinoembryonic antigen (ng/mL)	11.93	0–5
Glycated antigen 199 (U/mL)	37.95	0–19
Glycated haemoglobin HbA1c (%)	6.30	4.0–6.0

The patient was diagnosed with HCV-related compensated liver cirrhosis. Furthermore, by combining the endocrinology department physician’s suggestion, the patient’s clinical symptoms, serum cortisol, and 24 hour urine free cortisol test results, we excluded endogenous Cushing syndrome but not medical Cushing syndrome. She received hepatoprotective treatment with acetylcysteine and enzyme-lowering treatment with dicyclomine for approximately 5 days during his stay in our department while still taking oral SOF/VEL antiviral therapy. We discontinued all 3 drugs when we found that the clinical presentation was probably medically induced Cushing syndrome. We double-checked the instructions and found that neither acetylcysteine nor dicyclomine could be responsible for medically derived Cushing syndrome.

One month later, the patient was referred to a high-level hospital. The laboratory analysis results were as follows: serum ACTH 18.8 pg/mL at 8 am, and serum cortisol 0.7 µg/dL at 8 am. The aldosterone (standing), angiotensin II (standing), and renin (standing) levels were not significantly different. The patient’s clinical manifestations, hormone test results, and significantly reduced serum ACTH and cortisol levels were consistent with exogenous ICS due to corticosteroid intake. The patient had recently not taken glucocorticoids or unauthorized amounts of oral drugs, topical creams, sprays, or cosmetics. She developed medically induced Cushing syndrome after receiving direct anti-HCV treatment with SOF/VEL. The specialist considered that the patient’s serum cortisol and ACTH levels had fallen significantly below normal levels, rhythmicity had disappeared and there was hypothalamic–pituitary–adrenal axis suppression, requiring supplementation with small doses of glucocorticoids and dose reduction to restore normal function of the hypothalamic–pituitary–adrenal axis. The patient was administered oral hydrocortisone acetate tablets (10 mg) thrice daily.

**Figure 2.**
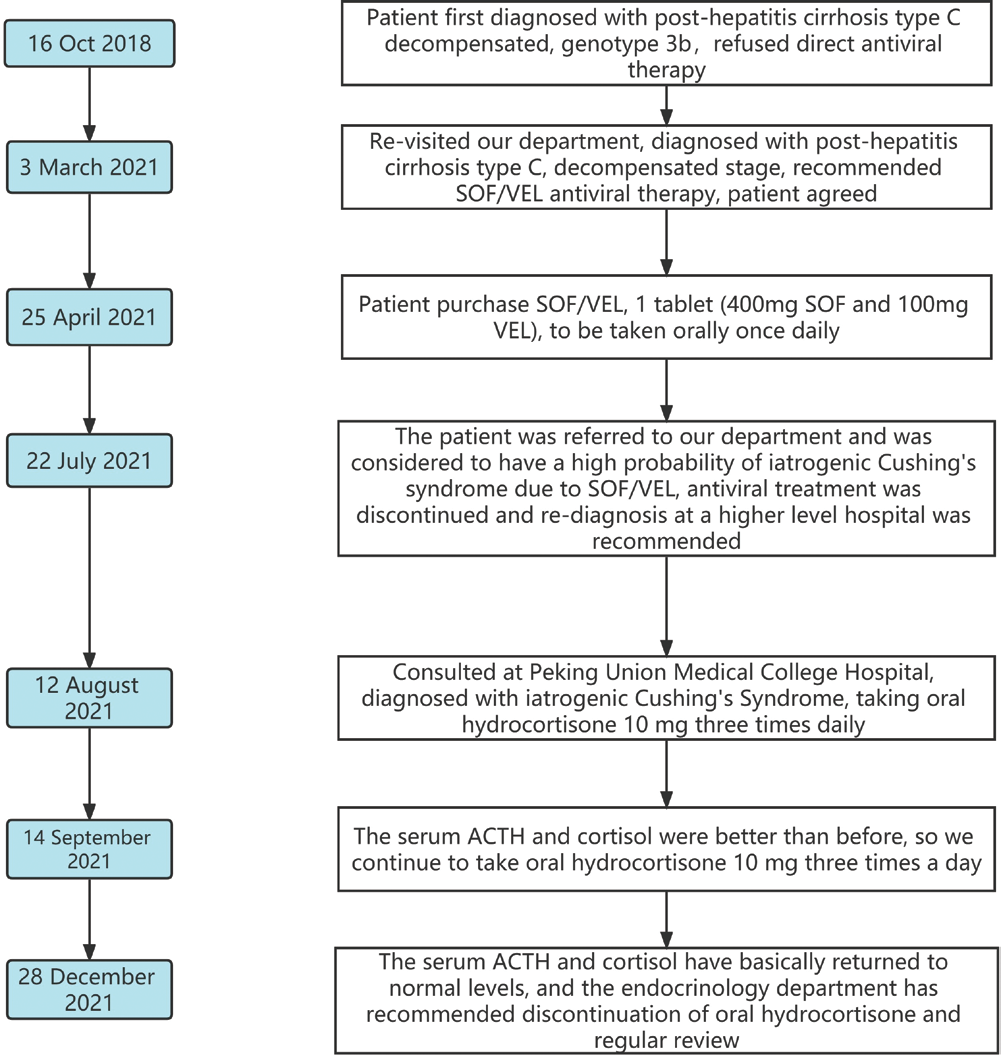
The flow of patient treatment and follow-up time.

On September 14, 2021, the patient was reexamined at our outpatient clinic after taking 10 mg of hydrocortisone acetate tablets orally 3 times a day for 1 month. The results showed that both serum ACTH and cortisol levels increased significantly (8 am–4 pm serum ACTH (pmol/L): 0.69–0.44; 8 am–4 pm serum cortisol (nmol/L): 14.06–10.43), indicating that the symptoms were greatly alleviated. The patient remained on hydrocortisone 10 mg orally 3 times daily and was followed up on December 28, 2021, with near-normal serum ACTH and cortisol values. The trends in hormonal changes are shown in Table 2. The patient also had slight clinical remission associated with Cushing syndrome compared with the previous one. An endocrinology consultation was requested, and the opinion indicated that hydrocortisone could be discontinued and reviewed regularly. The flow of patient treatment and follow-up time are shown in Figure 2.

**Table 2:** The trends of hormonal changes.

	July 22nd	September 14th	December 28th	Normal range
Cortisol at 0 (nmol/L)	11.2			240–619
Cortisol at 8 (nmol/L)	11.43	14.06	224.35	240–619
Cortisol at 16 (nmol/L)	19.54	10.43	130.38	<276
Adrenocorticotropic hormone at 0 (pmol/L)	0.22			1.6–13.9
Adrenocorticotropic hormone at 08 (pmol/L)	0.22	0.69	2.32	1.6–13.9
Adrenocorticotropic hormone at 16 (pmol/L)	0.22	0.44	1.31	1.6–13.9
24 h urinary free cortisol (nmol/24 h)	34.75	15.04		108–961
24 h urinary potassium (mmol/24 h)	56.6			51–102
24 h urinary sodium (mmol/24 h)	396.6			130–260
24 h urine calcium (mmol/24 h)	8.38			2.5–7.5
24 h urine chloride (mmol/24 h)	317.6			100–250
24 h urine phosphorus (mmol/24 h)	15.8			22–48
Estradiol 108 (pmol/L)	108			
Progesterone (nmol/L)	<0.38			
Prolactin (mIU/L)	354.9			
Follicle stimulating hormone (mIU/L)	6.57			
Testosterone (nmol/L)	<0.17			<0.56–2.46
Luteinizing hormone (mIU/mL)	6.6			

## 3. Discussion

ICS is a systemic adverse response to medication. It is most often found in patients with chronic supra-pathological dosages of glucocorticoid analogs,^[5]^ but less frequently with direct anti-hepatitis C drugs. Clinical signs include central obesity, purple lines, dorsocervical fat pads, muscular weakness, hypertension, easy bruising, moon face, and hypertrichosis.^[6]^ The age of the patient, size of the lesions, integrity of the epithelium, location of lesions, length of oral steroid treatment, and potency of the medicine are all characteristics linked to the development of IGS.^[6]^ Early detection is necessary to discontinue the culprit medicine as soon as possible. Depending on the severity of the condition, treatment involves long-term monitoring and the selection of suitable steroid hormone replacement therapy dosages. To the best of our knowledge, this is the first report of iatrogenic Cushing due to SOF/VEL. This case has not previously been reported in the literature.

SOF/VEL, a fixed-dose combination, was the first all-genotype DAA to be approved. Its efficacy and safety have been established in both clinical studies and real-world situations.^[7,8]^ The SOF/VEL regimen offers various benefits, including a smaller pill load, fewer possible medication interactions, and the ability to be used in patients with decompensated cirrhosis and renal failure.^[9]^ In 2 phase 3 randomized, double-blind, placebo-controlled clinical studies, the safety of SOF/VEL for the treatment of chronic hepatitis C was assessed.^[10]^ Side effects, such as headache, tiredness, diarrhea, and nausea were the most prevalent adverse events recorded.^[10]^ A recent retrospective analysis found that the most common side effects were stomach pain, tiredness, and pruritus.^[11]^ Headache (16.8%), fatigue (16.2%), nausea (11.8%), and insomnia (11.1%) were the most common adverse reactions observed in a 12-week study of Taiwanese patients with HCV-related decompensated liver cirrhosis treated with SOF/VEL. Grade 3 total bilirubin and alanine aminotransferase elevations occurred in 9 (0.5%) and 2 (0.1%) patients, respectively.^[2]^ Wong et al^[3]^ found no serious adverse events observed with the use of SOF/VEL for real-world chronic hepatitis C genotype 3 cohorts, except for myositis in 1 patient. The presence of ICS as a side effect was not mentioned in these studies.

In this case, the patient first presented with medical-derived Cushing syndrome due to SOF/VEL, which improved with steroid hormone supplementation and still required further follow-up. Furthermore, the patient had a history of psoriasis, which is an inherited disorder of the immune system most often seen in the skin and joints, but is also associated with cardiovascular, metabolic, and neuropsychiatric effects. These findings indicated the presence of underlying immune disorders. What intrigues us is that the patient had never been treated with other steroid-containing medications, as we have repeatedly confirmed. The cause of these side effects remains unclear. The patient reported that she started noticing changes in her face when she was taking the oral anti-disease liver drug SOF/VEL for 2 months, but the changes were not very noticeable. Therefore, the patient was not concerned and did not undergo further investigations. Early review should have led to the early detection of Cushing syndrome, and early discontinuation of the potentially offending drug should have mitigated the extent to which this adverse event occurred, but whether it could have been avoided is unclear. Therefore, further studies on the occurrence of this adverse event in a large cohort are needed.

The patient’s hepatitis C nucleic acid quantification was negative after nearly 3 months of antiviral therapy. However, not all patients are successfully treated with antiviral therapies. Therefore, in the event of an adverse event, the potentially offending drug should be discontinued immediately, and the HCV viral load reassessed before deciding on the next treatment step. In the event of serious adverse events and discontinuation of the drug, finding an alternative direct antiviral agent remains a major clinical challenge. Glicaprevir/Pibrantasvir is another option for compensated HCV-associated cirrhosis if the patient fails SOF/VEL. The patient was then followed up, and his serum ACTH and cortisol levels had almost returned to normal after 4 months of oral low-dose hydrocortisone, and his clinical symptoms had improved slightly.

This study highlights the importance of collaboration between hepatologists and endocrinologists in the co-management of complications and aims to improve early recognition of this rare adverse event by hepatologists and primary care physicians treating HCV, allowing early discontinuation of DAA therapy if a similar adverse event recurs.

## Author contributions

**Funding acquisition:** Tianling Shang, Jianjie Huang, Zhixin Tu.

**Investigation:** Yan Wang, Yujin Han, Yang Wang.

**Resources:** Qinglong Jin.

**Supervision:** Tianling Shang, Jianjie Huang.

**Validation:** Xiaoyu Wen, Qinglong Jin.

**Writing – original draft:** Bo Ma.

**Writing – review & editing:** Bo Ma.
